# HELLS serves as a poor prognostic biomarker and its downregulation reserves the malignant phenotype in pancreatic cancer

**DOI:** 10.1186/s12920-021-01043-5

**Published:** 2021-07-27

**Authors:** Feng-Jiao Wang, Yan-Hua Jing, Chien-Shan Cheng, Zhang-Qi Cao, Ju-Ying Jiao, Zhen Chen

**Affiliations:** 1 Department of Integrative Oncology, Fudan University Shanghai Cancer Center, 270 Dong An Road, Shanghai, 200032 China; 2 Department of Oncology, Shanghai Medical College, Fudan University, Shanghai, 200032 China

**Keywords:** SMARCA, HELLS, Pancreatic cancer, Prognostic value, Data mining analysis

## Abstract

**Background:**

SMARCAs, belonged to SWI/SNF2 subfamilies, are critical to cellular processes due to their modulation of chromatin remodeling processes. Although SMARCAs are implicated in the tumor progression of various cancer types, our understanding of how those members affect pancreatic carcinogenesis is quite limited and improving this requires bioinformatics analysis and biology approaches.

**Methods:**

To address this issue, we investigated the transcriptional and survival data of SMARCAs in patients with pancreatic cancer using ONCOMINE, GEPIA, Human Protein Atlas, and Kaplan–Meier plotter. We further verified the effect of significant biomarker on pancreatic cancer in vitro through functional experiment.

**Results:**

The Kaplan–Meier curve and log-rank test analyses showed a positive correlation between SMARCA1/2/3/SMARCAD1 and patients’ overall survival (OS). On the other hand, mRNA expression of SMARCA6 (also known as HELLS) showed a negative correlation with OS. Meanwhile, no significant correlation was found between SMARCA4/5/SMARCAL1 and tumor stages and OS. The knockdown of HELLS impaired the colony formation ability, and inhibited pancreatic cancer cell proliferation by arresting cells at S phase.

**Conclusions:**

Data mining analysis and cell function research demonstrated that HELLS played oncogenic roles in the development and progression of pancreatic cancer, and serve as a poor prognostic biomarker for pancreatic cancer. Our work laid a foundation for further clinical applications of HELLS in pancreatic cancer.

## Introduction

The switch/sucrose nonfermenting complex (SWI/SNF) is an ATPase-dependent multisubunit complex modulating gene expression involved in chromatin remodeling and transcriptional regulation [1]. The whole-exome sequencing data, including 18 neoplastic entities from 24 published studies, showed widespread SWI/SNF mutations among diverse human cancers (20%), excessive deleterious mutations, and an overall frequency approaching TP53 mutations [2]. Recent studies investigated the crucial implication of the SWI/SNF protein complex in the initiation and dedifferentiation of various of neoplasms, which might attribute to its regulation of differentiation and cell proliferation through their enrichment promoters and enhancers active genes [3].

Several SWI/SNF2 family members are known by a SMARCA (SWI/SNF-related Matrix-associated, Actin-dependent Regulator Chromatin Group A) class or chromatin subgroup remodeler. SMARCA class of chromatin remodeling genes (SMARCAs) play a crucial role in chromatin remodeling, especially in double-strand damage repair [4]. SMARCAs are further made up of SMARCA1, SMARCA2, SMARCA3, SMARCA4, SMARCA5, HELLS, SMARCAD1, and SMARCAL1 [5]. Previous studies purposed the SMARCA family members’ carcinogenic role in several undifferentiated and differentiated cancers, including lung cancer and renal cell cancer [4]. However, the expression patterns and the exact roles of SMARCAs in pancreatic cancer remained unclear. In this study, the protein and mRNA expression, prognostic values, and potential functions of different SMARCA family members in pancreatic cancer were systematically explored.

## Materials and methods

### ONCOMINE database analysis

ONCOMINE database (www.oncomine.org) is a publicly accessible online cancer microarray database to collect, standardize, analyze, and deliver cancer transcriptome data to the biomedical research community [6]. The expression levels of SMARCA gene family members in different types of cancer tissues and their adjacent normal samples were identified using ONCOMINE database. The differences were compared by students’ *t*-test. The threshold was restricted as follows: *P* value: 0.01, fold change: 2, gene rank: 10%, data type: mRNA.

### GEPIA dataset analysis

Gene Expression Profiling Interactive Analysis (GEPIA) (http://gepia2.cancer-pku.cn/) is a developed interactive web server for estimating the RNA sequencing expression data, based on 9,736 tumors and 8,587 normal samples from the Cancer Genome Atlas (TCGA) and Genotype-Tissue Expression (GTEx) dataset projects. The UCSC Xena project (http://xena.ucsc.edu/) has recomputed all raw RNA-Seq data based on a standard processing pipeline, thus minimizing differences from distinct sources and making such data more compatible. In addition, GEPIA provides essential interactive and customizable functions, including differential expression analysis, profiling plotting, patient survival analysis, similar gene detection, correlation analysis, and dimensionality reduction analysis [7].

### Kaplan–Meier plotter

The prognostic value of mRNA expression of SMARCAs in pancreatic cancers was evaluated using an open online database, Kaplan–Meier Plotter (http://kmplot.com/analysis/). This database provided gene expression data and survival information of patients with liver cancer and many other cancer types, such as breast cancer, ovarian cancer, lung cancer, and gastric cancer [8–10]. Patients were divided into high and low expression groups according to the median values of mRNA expression and validated by Kaplan–Meier survival curves with a hazard ratio (HR) with 95% confidence intervals (CIs). A log-rank *P *value < 0.05 was considered statically significant.

### Immunohistochemistry staining

The Human Protein Atlas (HPA) (https://www.proteinatlas.org/) is a website that aims to map human proteins in cells, tissues, and organs by utilizing various omics technologies that contain immunohistochemistry-based protein expression, mass spectrometry-based proteomics, transcriptomics, and systems biology [11]. In this study, different SMARCA members’ expression between pancreatic cancer and normal tissues was verified by immunohistochemistry image.

### Functional enrichment analysis

Metascape (http://metascape.org) is a free gene-list analysis tool for gene annotation and analysis and an automated meta-analysis tool to understand common and unique pathways within a group of orthogonal target-discovery studies [12]. In this study, the neighboring genes based on TCGA and GTEx expression datasets with similar expression pattern with SMARCA family members were detected using GEPIA, respectively. The selected correlate genes were further used to conduct pathway and process enrichment analysis by Metascape. For this, the Gene Ontology (GO) enrichment analysis, including biological processes (BP), cellular components (CC), and molecular functions (MF) were used for predicting the functional roles of SMARCAs mutations and similar genes associated with SMARCAs mutations. Kyoto Encyclopedia of Genes and Genomes pathways (KEGG) analyzed the SMARCA family members-related pathways and closely related neighbor genes of SMARCAs mutations. A *P* value cut-off < 0.01, a minimum overlap of 3, and a minimum enrichment factor of 1.5 were considered as statistically significant.

### Western blotting

Cells were collected and lysed in cold RIPA buffer supplemented with phenylmethanesulfonyl fluoride (PMSF; Beyotime, China) and centrifuged at 14,000 rpm and 4 °C for 15 min. Total protein concentrations were determined with a BCA protein assay kit (Beyotime, China). Equal amounts of denatured protein samples were separated by electrophoresis using 10% SDS-PAGE gel fast preparation kits (Epizyme, China), and then transferred onto 0.45 μm polyvinylidene difluoride (PVDF; Millipore, USA) membranes. Subsequently, the membranes were blocked with 5% BSA diluted in Tris-buffered saline with 0.1% Tween-20 (TBST) at room temperature. Then, the PVDF membranes were incubated with primary antibodies against β-actin (1:1000; Proteintech) and HELLS (1:1000; Proteintech) at 4 °C overnight. The next day, the primary antibody was washed three times with TBST and incubated with HRP-conjugated secondary antibodies at room temperature for 1 h.

### Cell culture and HELLS knockdown

The human pancreatic cancer cell lines were obtained from the American Type Culture Collection (ATCC, USA), and cultured in Dulbecco’s modified Eagle’s medium (DMEM) supplemented with 10% fetal bovine serum (FBS), 100 μg/ml penicillin and 100 μg/ml streptomycin in a humidified incubator at 37 °C under an atmosphere with 5% CO2.

The packaged lentivirus containing shRNAs targeting HELLS (shHELLS-1, 5′-AACAAGGCGATAAACAACAAC-3′; shHELLS-2, 5′-TTCTACAGGGATATTCACTTC-3′; shHELLS-3, 5′-AATTGTTTCTTTCTCACTGGA-3′) and a negative control sequence (shHELLS-con, 5′-TTCTCCGAACGTGTCACGT-3′) were designed by GENE (Shanghai, China). Cells were seeded in 24-well plates and cultured for 24 h. Then, shRNAs were transfected using transfection reagent provided by GENE. After incubation of 72 h to 80% confluence, cells were selected in puromycin and the knockdown efficiency of HELLS was confirmed by quantitative real-time PCR (qRT- PCR) and western blotting.

### RNA extraction and qRT- PCR

Total RNA was extracted from cells using RNAiso Plus reagent (Takara, Tokyo, Japan). The cDNA was synthesized using Takara PrimeScript™ RT Master Mix. qRT- PCR was performed with TB Green® Premix Ex Taq™ using an ABI 7900HT Real-Time PCR system (Applied Biosystems, CA, USA). The relative gene mRNA expressions were calculated by 2^− ΔΔCt^ method with respect to GAPDH. The primer sequences are listed in Table 1.

**Table 1:** The sequences of primers

Primers	Sequences (5′–3′)
HELLS Forward	ACTCCTCCTCTACTAATCTCTG
HELLS Reverse	GGCTGACCATTACACTTCC
GAPDH Forward	GCACCGTCAAGGCTGAGAAC
GAPDH Reverse	TGGTGAAGACGCCAGTGGA

### Cell proliferation assay

A cell counting kit-8 (CCK-8; DOJINDO, China) was used to measure cell proliferation. In brief, cell suspensions at a density of 3 × 10^3^ cells per 200 μl were seeded in 96-well plates and incubated for 0, 24, 48, 72, 96 h. After the indicated incubation times, the medium was removed and a CCK-8 DMEM solution (10 µl of CCK-8 in 100 µl of DMEM) was added into each well and incubated at 37 °C for an additional 2 h. The absorbance of each well at 450 nm were measured using a microplate reader.

### Colony formation assay

A total of 500 cells per well were seeded in 6-well plates and incubated for 14 days. After that, cell colonies were washed with phosphate-buffered saline (PBS) three times, fixed with 4% paraformaldehyde for 30 min, and further stained with 0.1% crystal violet solution for 15 min. Cell visible colonies containing more than 50 cells were captured by mobile camera.

### Cell cycle analysis

The cell cycle was examined by flow cytometric analysis with propidium iodide (PI)/RNase staining kit (Beyotime, China). Cell suspensions at a density of 3 × 10^5^ cells/well were seeded in 6-well plates and incubated overnight. After cycle synchronization by serum starvation for 24 h, cells were cultured in regular DMEM for 48 h. Then, cells were washed in cold PBS and centrifuged at 1000×*g* for 5 min, and resuspended in ice-cold 70% ethanol for at least 2 h on ice. Centrifuged, decant and washed again in cold PBS. About 1 × 10^6^ cells were resuspended in 0.5 ml working solution per sample (500 µl of assay buffer mixed 25 µl of PI solution and 2.5 µl RNase solution), and kept for 30 min at 37 °C and 4 °C in the dark, respectively.

### Statistical analysis

Statistical analysis was performed by GraphPad Prism (version 7.0a), and the results are presented as the means ± SD. Two-tailed unpaired Student’s *t*-test, and one-way or two-way ANOVA were used to evaluate the data. *P*-value less than 0.05 was considered statistically significant.

## Results

### Transcriptional levels and protein expression of SMARCAs in pancreatic cancer patients

**Fig. 1:**
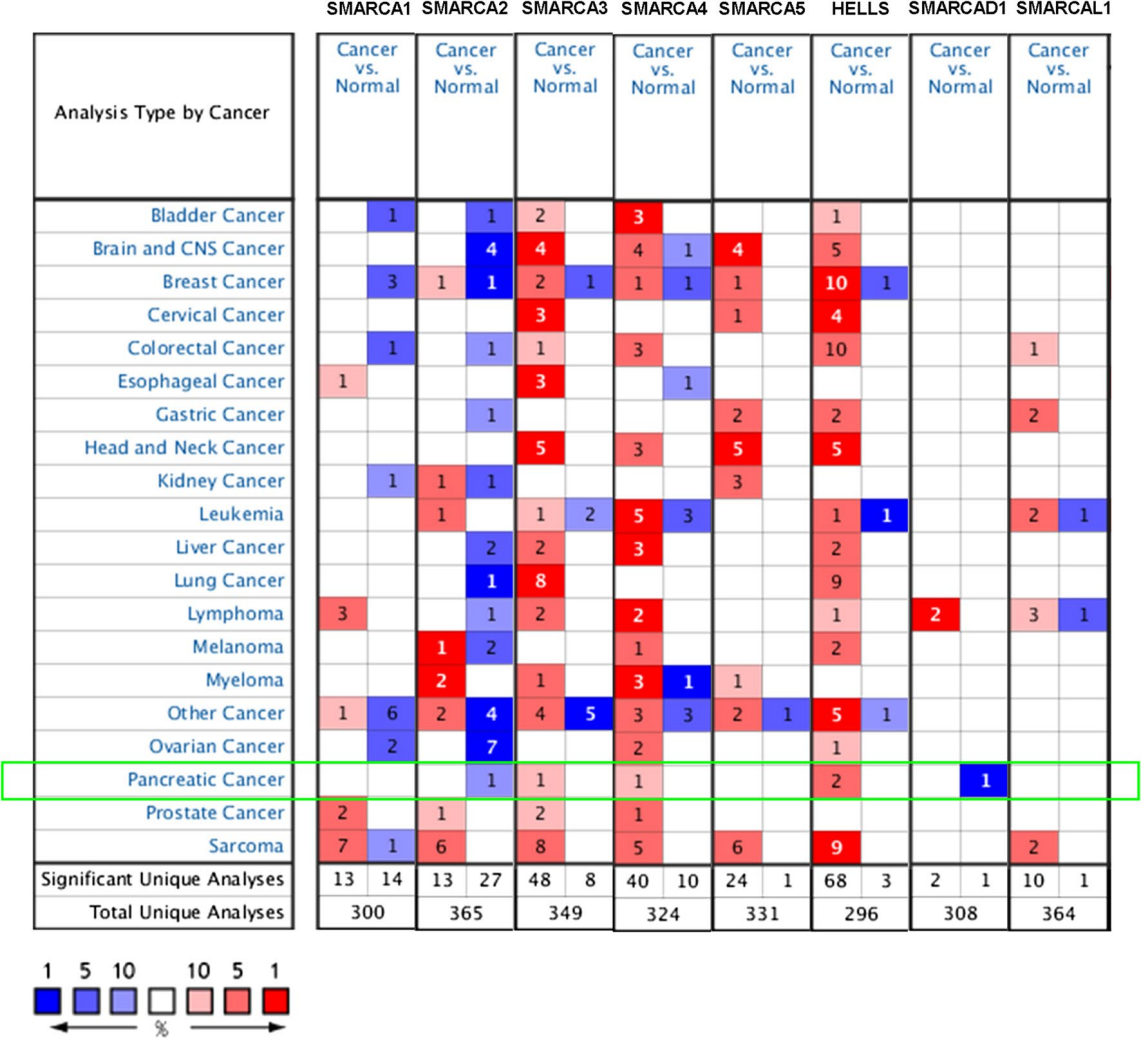
The transcriptional expression of SMARCAs in different types of cancers (ONCOMINE database). The differences of transcription levels were compared by students’ *t* test. The threshold was restricted as follows: *P* value: 0.01, fold change: 2, gene rank: 10%, data type: mRNA

Eight SMARCA family members have been identified in mammalian cancers. The different transcriptional levels of SMARCAs in pancreatic cancer and normal samples were analyzed by ONCOMINE database (Fig. 1; Table 2). Results revealed that the mRNA levels of SMARCA3, SMARCA4, HELLS were significantly upregulated in patients with pancreatic cancer, while the mRNA levels of SMARCA2 and SMARCAD1 were downregulated (Fig. 1). In Table 2, the results showed that the transcription levels of SMARCA3 were significantly higher in patients with pancreatic cancer in Buchholz’s dataset [13]. SMARCA3 was overexpressed in pancreatic ductal adenocarcinoma compared to the normal tissues, with a 2.522 fold-change. In Logsdon’s dataset [14], the transcription levels of SMARCA4 were significantly higher in patients with pancreatic adenocarcinoma, with a fold change of 2.257 and a *P* value of 7.16E−4. HELLS was highly expressed in two datasets [15, 16]. HELLS was overexpressed in pancreatic ductal adenocarcinoma compared with that in the normal samples in Grutzmann’s dataset [15], with a fold change of 2.596 and a *P* value of 0.005. In Pei’s dataset [16], HELLS was overexpressed in pancreatic cancer with a fold change of 2.386 and a *P* value of 3.50E−8. However, SMARCA2 was found significantly decreased in patients with pancreatic cancer compared with that in the normal samples in Buchholz’s dataset [13], with a fold change of − 6.555 and a *P* value of 0.009.

**Table 2:** SMARCAs transcription levels between pancreatic cancer and normal tissues (ONCOMINE)

	Types of pancreatic cancer versus normal pancreatic tissue	Fold change	*P* value	t test	References
SMARCA2	Pancreatic ductal adenocarcinoma	− 6.555	0.009	3.107	Buchholz Pancreas [13]
SMARCA3	Pancreatic ductal adenocarcinoma	2.522	0.008	3.688	Buchholz Pancreas [13]
SMARCA4	Pancreatic adenocarcinoma	2.257	7.16E−4	4.459	Logsdon Pancreas [14]
HELLS	Pancreatic ductal adenocarcinoma	2.596	0.005	2.867	Grutzmann Pancreas [15]
	Pancreatic carcinoma	2.386	3.50E−8	6.352	Pei Pancreas [16]

Order by Over-expression: Fold Change; *P* value < 0.01; Fold Change > 2; Gene Rank: Top 10%

**Fig. 2:**
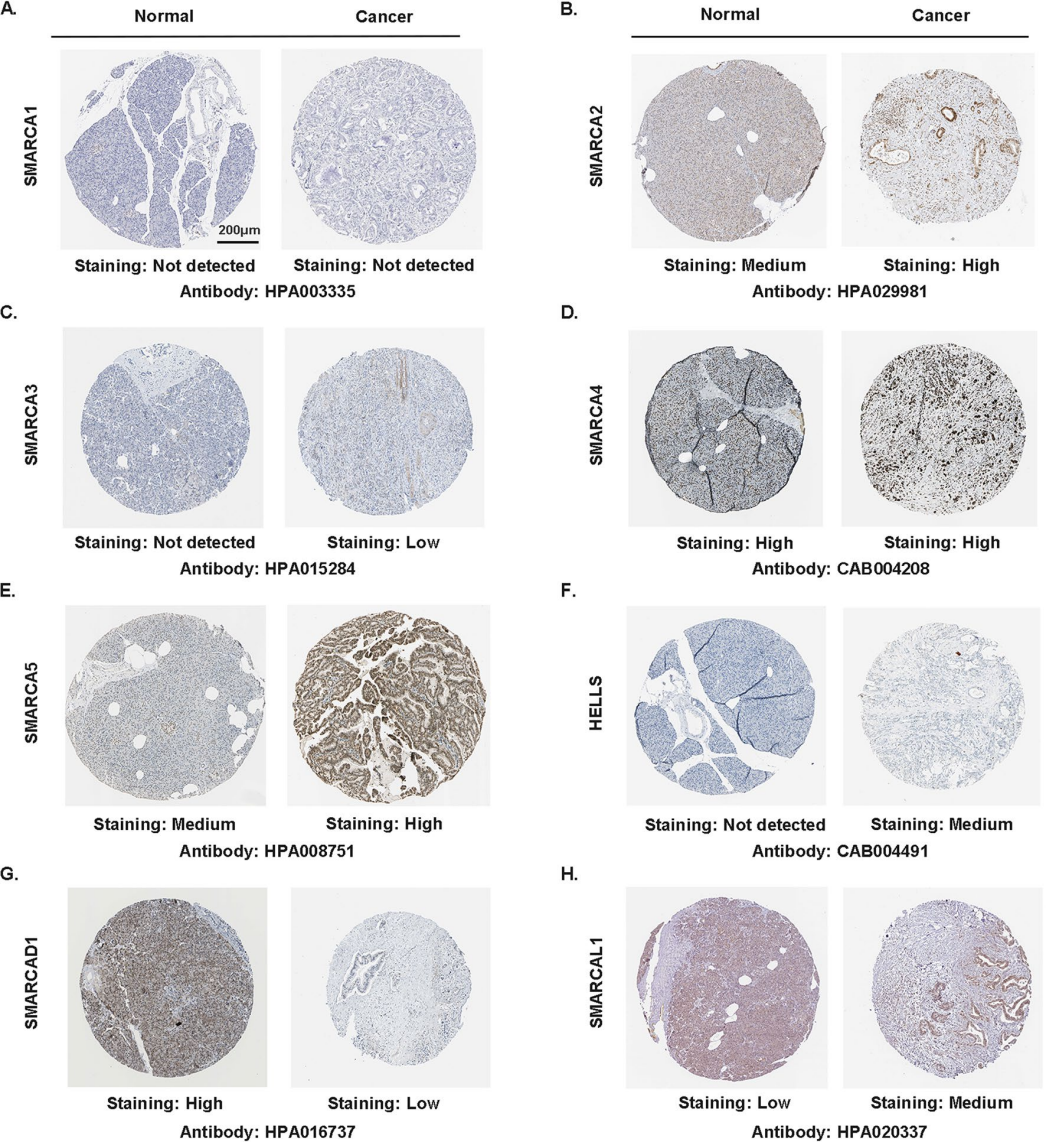
Protein expression of SMARCAs obtained from the Human Protein Atlas (HPA) online database (scale bar, 200 μm). Staining was divided into not detected, low, medium and high based on the intensity of staining. **A **SMARCA1 protein was not detected in normal tissues and pancreatic cancer tissues; **B, E, H** SMARCA2/5 and SMARCAL1 protein were expressed medium or low in normal tissues, while high or medium expression were observed in pancreatic tissues; **C, F** SMARCA3 and HELLS protein were not observed in normal pancreatic tissues, whereas low and medium expression were detected in pancreatic cancer tissues, respectively; **D** SMARCA4 protein was expressed high both in normal tissues and pancreatic cancer tissues; **G** SMARCAD1 protein was lower in pancreatic cancer tissues compared to normal tissues

Additionally, after examining the transcriptional levels of SMARCAs in pancreatic cancer, we explored the protein expression of SMARCAs using the HPA online database. We found that SMARCA3 and HELLS proteins were not observed in normal pancreatic tissues, whereas low and medium expressions were detected in pancreatic cancer tissues, respectively (Fig. 2C, F). However, lower protein expressions of SMARCAD1 was observed in pancreatic cancer tissues compared to normal tissues (Fig. 2G). SMARCA2/5 and SMARCAL1 protein were expressed medium or low in normal tissues, while high or medium expression was observed in pancreatic tissues (Fig. 2B, E, H). High protein expression of SMARCA4 was observed both at normal pancreatic tissues and cancer tissues (Fig. 2D), while SMARCA1 was not detected in both of them (Fig. 2A).

### Association between the mRNA expression of SMARCAs and the clinicopathological parameters of patients with pancreatic cancer

**Fig. 3:**
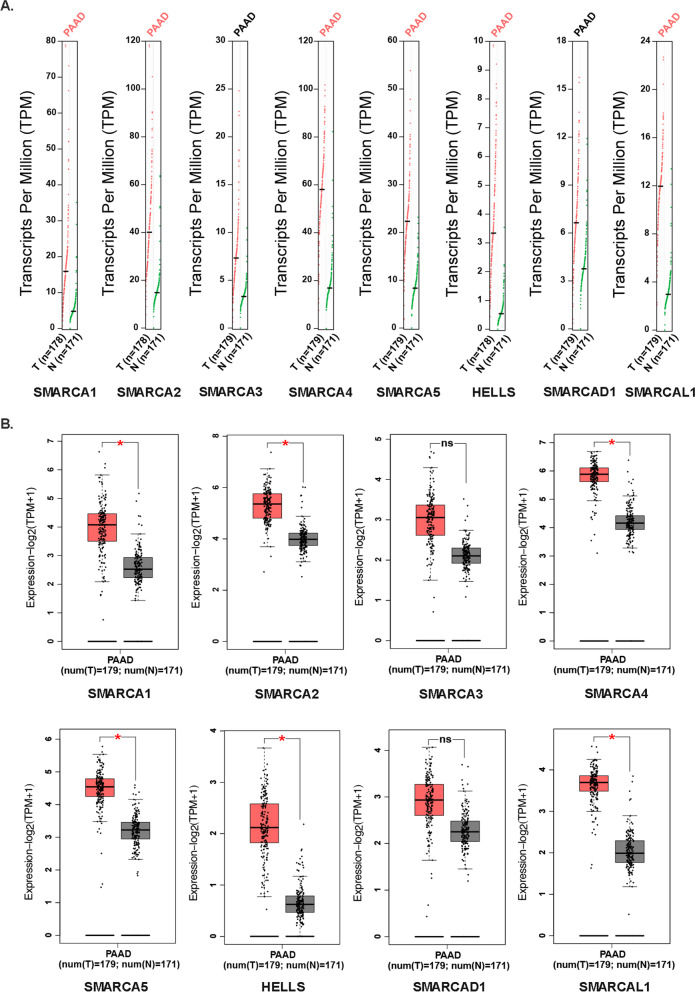
The expression of SMARCAs between pancreatic cancer tissues and normal tissues (GEPIA). **A** The transcripts per million (TPM) levels of SMARCAs between pancreatic cancer tissues and normal tissues; **B** The mRNA levels of SMARCAs between pancreatic cancer tissues and normal tissues

**Fig. 4:**
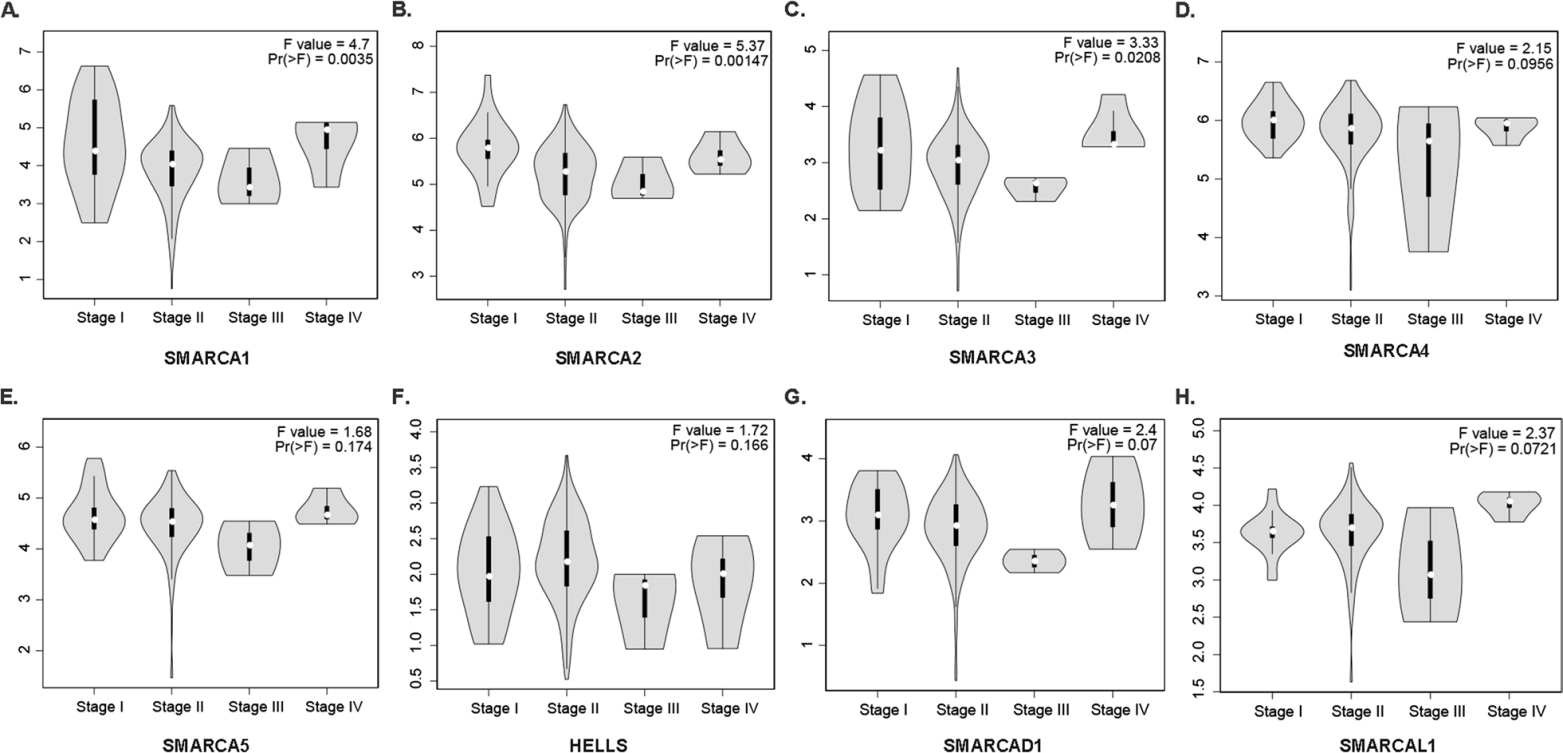
Correlation between the mRNA expression of SMARCAs and tumor stages in patients with pancreatic cancer (GEPIA). **A**–**C** The expression levels of SMARCA1, SMARCA2, and SMARCA3 were remarkably correlated with the tumor stages (*P* < 0.05); **D**–**H** The expression levels of SMARCA4, SMARCA5, HELLS, SMARCAD1, and SMARCAL1 did not differ significantly (*P* > 0.05)

We compared the mRNA Levels of SMARCA family members between pancreatic cancer tissues and normal tissues using the GEPIA. In our study, the results demonstrated the higher transcripts per million (TPM) levels of SMARCA1, SMARCA2, SMARCA4, SMARCA5, HELLS, and SMARCAL1 in pancreatic cancer samples than that in normal control (*P* < 0.05), which suggests that the above six genes possessed more transcripts in pancreatic cancer tissues (Fig. 3A). Consistent with the mRNA levels of SMARCA1, SMARCA2, SMARCA4, SMARCA5, HELLS, and SMARCAL1, a significant difference was found in patients with pancreatic cancer (*P* < 0.05), and the mRNA levels of SMARCA3 and SMARCAD1 displayed no significant difference between the pancreatic cancer group and the control group (*P* > 0.05) (Fig. 3B). Subsequently, we analyzed the correlation between the mRNA expression of SMARCAs and tumor stages in pancreatic cancer. The mRNA expression levels of SMARCA1, SMARCA2, and SMARCA3 were remarkably correlated with the tumor stages (*P* < 0.05) (Fig. 4A–C), whereas the mRNA expression of SMARCA4, SMARCA5, HELLS, SMARCAD1, and SMARCAL1 did not differ significantly (*P* > 0.05) (Fig. 4D–H).

### Prognostic value of mRNA expression of SMARCAs in pancreatic cancer

**Fig. 5:**
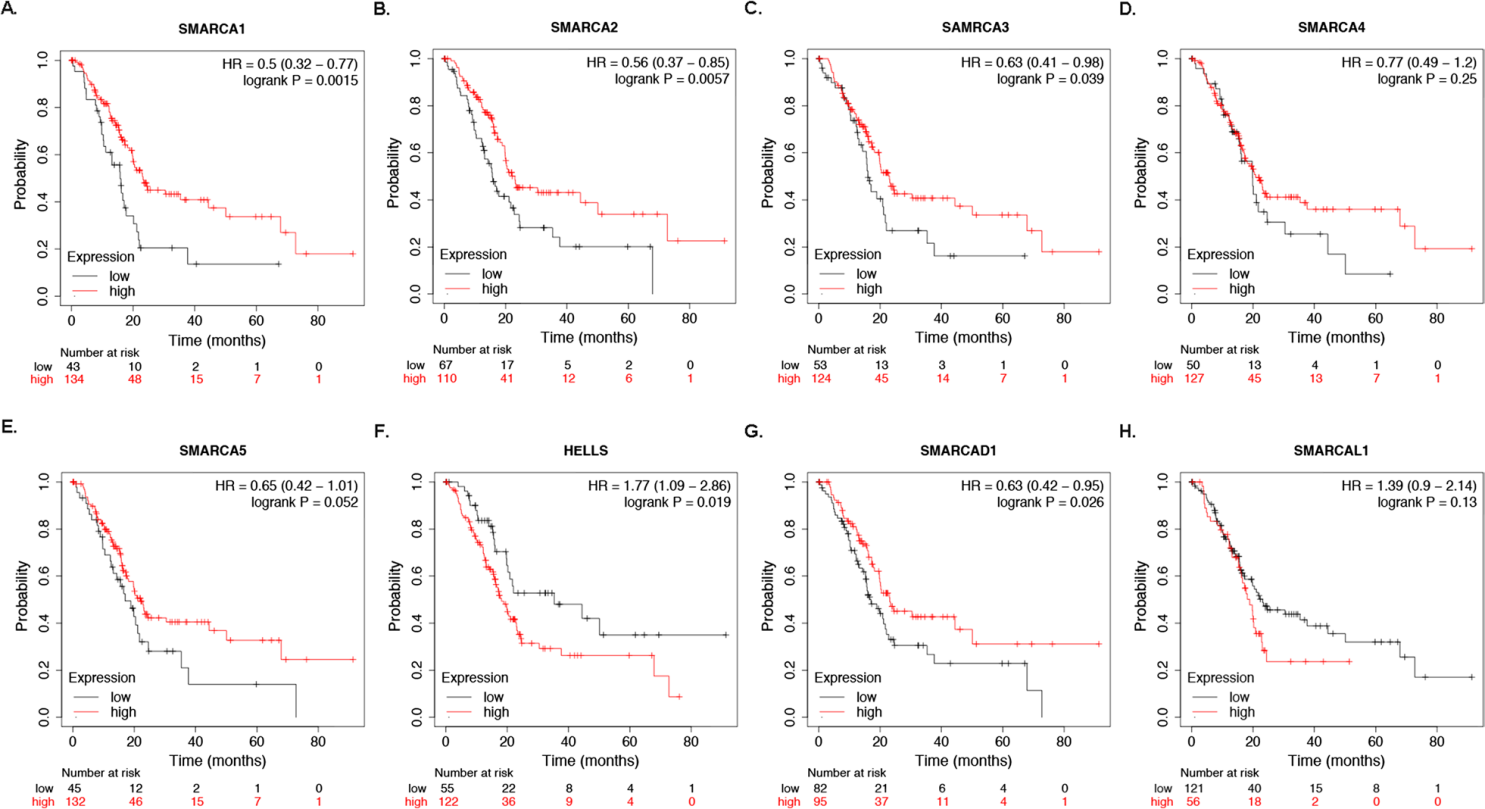
Prognostic value of mRNA expression of SMARCAs in pancreatic cancer (Kaplan–Meier Plotter). **A**–**C**, **G** The mRNA expression of SMARCA1, SMARCA2, SMARCA3 and SMARCAD1 were significantly correlated with the longer OS of pancreatic cancer patients; **F** The high mRNA expression of HELLS was significantly associated with shorter OS (*P* < 0.05);** D, E, H** The mRNA expression of SMARCA4, SMARCA5, and SMARCAL1 showed no significant correlation with the prognosis of pancreatic cancer (*P* > 0.05)

Further, we explored the potential value of SMARCAs in the survival of patients with pancreatic cancer using Kaplan–Meier Plotter tools. The Kaplan–Meier curve and log-rank test analyses showed that mRNA expression of SMARCA1, SMARCA2, SMARCA3, HELLS, and SMARCAD1 were significantly correlated with patients’ OS (Fig. 5A–C, F, G). To be more specific, the high mRNA levels of SMARCA1 (HR = 0.5, 95% CI 0.32–0.77, and *P* = 0.0015), SMARCA2 (HR = 0.56, 95% CI 0.37–0.85, and *P* = 0.0057), SMARCA3 (HR = 0.63, 95% CI 0.41–0.98, and *P* = 0.039) and SMARCAD1 (HR = 0.63, 95% CI 0.42–0.95, and *P* = 0.026) may contribute to favorable prognosis of pancreatic cancer (*P* < 0.05), while the mRNA expression of HELLS was negatively correlated with patients’ OS (HR = 1.77, 95% CI 1.09–2.86, and *P* = 0.019). However, the mRNA expression of SMARCA4, SMARCA5, and SMARCAL1 showed no significant correlation with the prognosis of pancreatic cancer (*P* > 0.05) (Fig. 5D, E, H). The above results indicated that high mRNA expressions of SMARCA1/2/3 and SMARCAD1, or low mRNA levels of HELLS were significantly associated with longer OS, which may be exploited as potential prognostic biomarkers for pancreatic cancer.

### Predicted functions and pathways of alterations in SMARCAs and their similar genes in pancreatic cancer

**Fig. 6:**
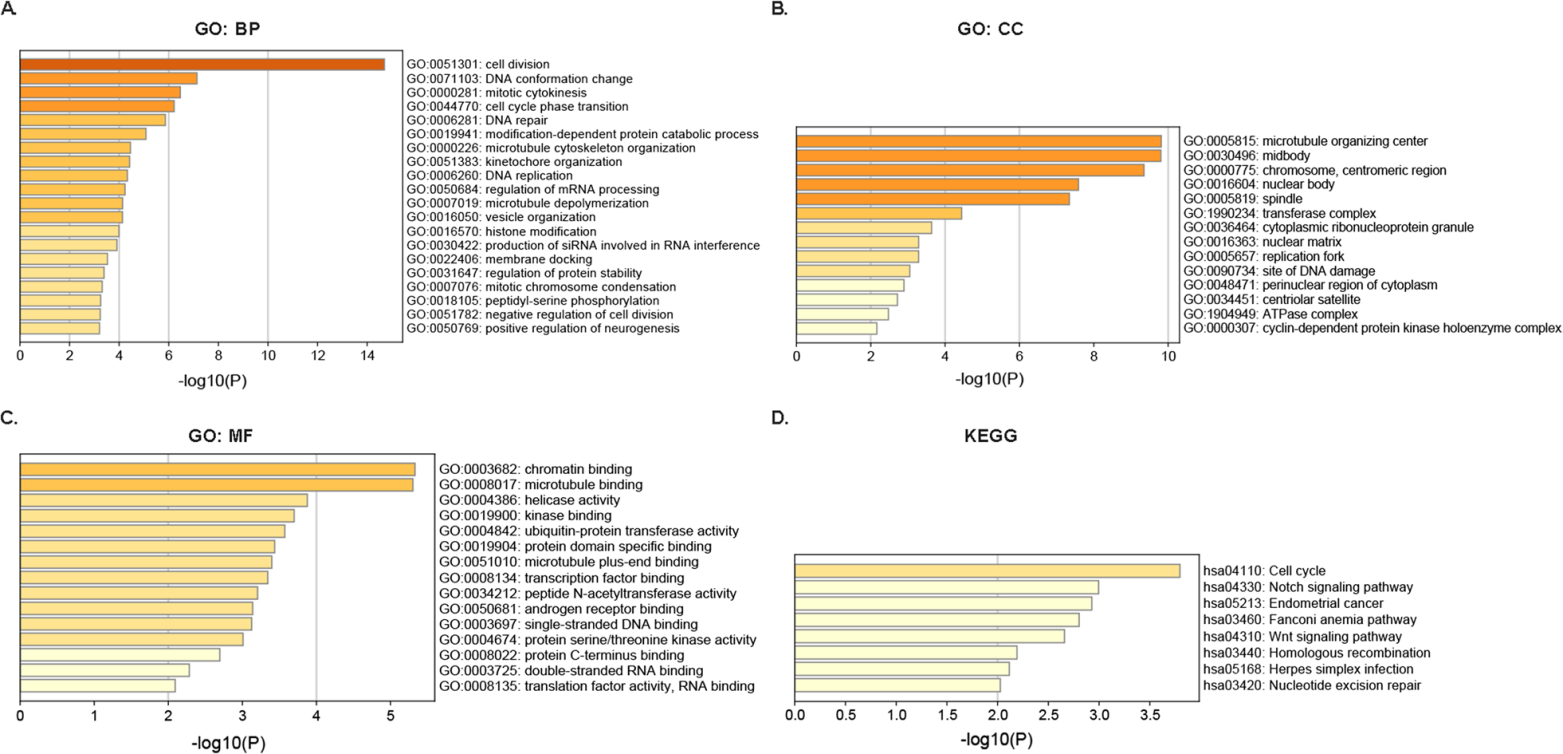
The functional enrichment analysis of SMARCAs in pancreatic cancer patients (Metascape). **A** Biological processes (BP). **B** Cellular components (CC). **C** Molecular functions (MF). **D** Kyoto Encyclopedia of Genes and Genomes pathways (KEGG) Analysis. A *P* value cut-off < 0.01, a minimum overlap of 3, and a minimum enrichment factor of 1.5 were considered as statistically significant. All enriched terms colored by *P* value

A total of 240 similar genes based on TCGA and GTEx expression datasets were detected by GEPIA, which were significantly correlated with SMARCAs alterations. The functions and pathways of alterations in SMARCAs and their similar genes were conducted by analyzing GO and KEGG in Metascape. The results showed that BP, such as GO: 0051301 (cell division), GO: 0071103 (DNA conformation change), GO: 0000281 (mitotic cytokinesis), and GO: 0044770 (cell cycle phase transition) were significantly regulated by the SMARCAs alterations in pancreatic cancer (Fig. 6A). CC, including GO: 0005815 (microtubule organizing center), GO: 0030496 (midbody), GO: 0000775 (chromosome, centromeric region), GO: 0016604 (nuclear body) and GO: 0005819 (spindle) were significantly associated with the SMARCAs mutations (Fig. 6B). In addition, SMARCAs alterations prominently affected the MF, including GO: 0003682 (chromatin binding) and GO: 0008017 (microtubule binding) (Fig. 6C). Among the KEGG pathways, hsa04110 (Cell cycle) and hsa04330 (Notch signaling pathway) were prominently involved in the tumorigenesis and pathogenesis of pancreatic cancer (Fig. 6D).

### Downregulation of HELLS suppresses pancreatic cancer cells proliferation, colony formation, and affects cell cycle distribution

**Fig. 7:**
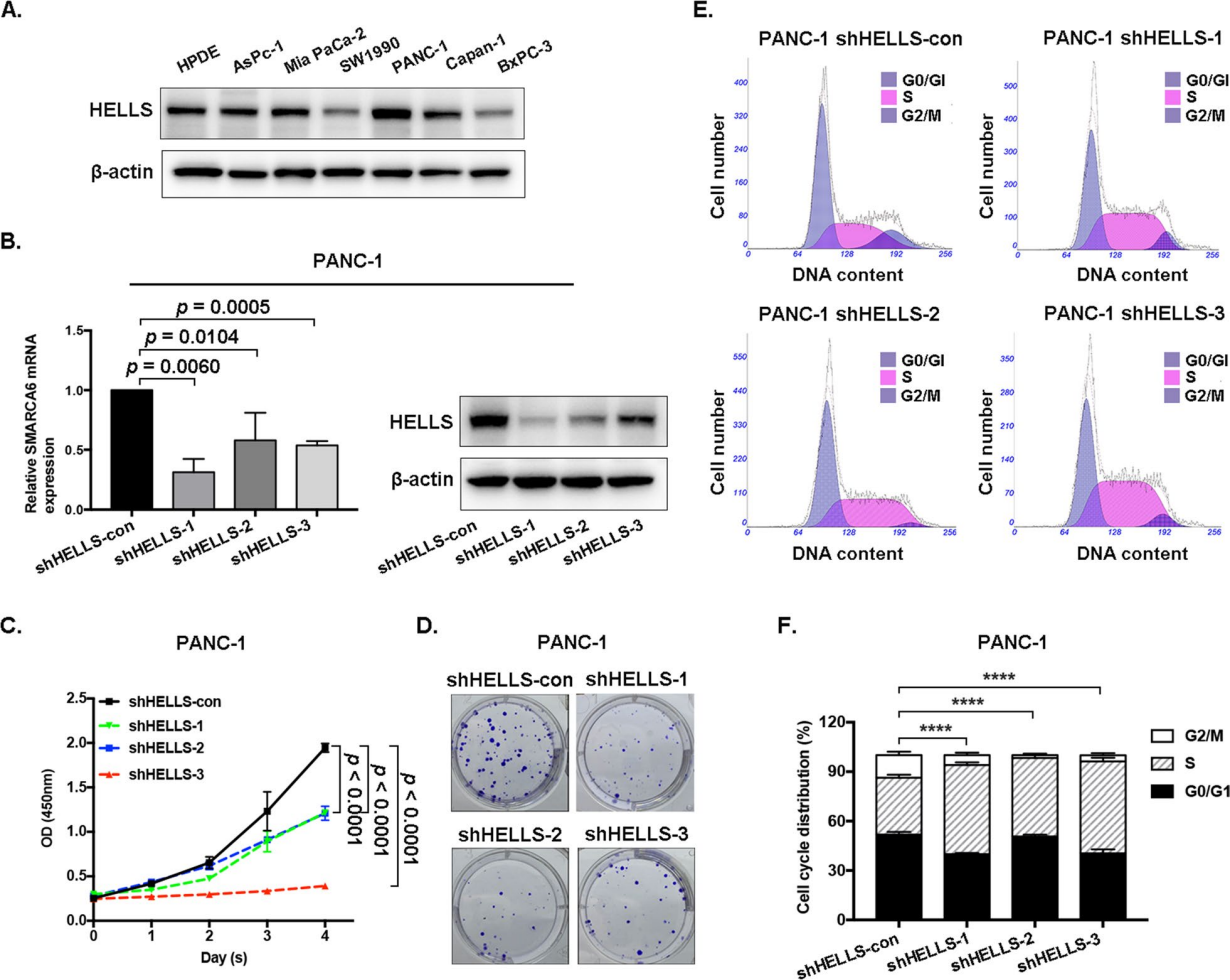
Downregulation of HELLS suppresses pancreatic cancer cells proliferation, colony formation, and affects cell cycle distribution. **A** The expression of HELLS in pancreatic cancer cell lines and normal human pancreatic ductal epithelial cells (HPDE). The grouping of blots cropped from different parts of the same gel, or from different gels, fields, or exposures was divided with white space. **B** Verification at mRNA and protein levels of shHELLS in PANC-1 cells. The grouping of blots cropped from different parts of the same gel, or from different gels, fields, or exposures was divided with white space. **C**, **D** Downregulation of HELLS significantly inhibited cell proliferation, and impaired the ability of the colony formation (*P* < 0.05). **E** Cell cycle distribution under different shHELLS analyzed by flow cytometry. **F** A significant increase in S fraction in shHELLS group compared with the shHELLS-con group (****P = 0.0001)

Notably, among the SMARCA family members, data mining analysis showed that mRNA and protein levels of HELLS are negatively correlated with outcomes in pancreatic cancer patients, which is highly in accordance with its oncogenic roles in previous literature. According to the abovementioned bioinformatic analysis results, HELLS could serve as a potential prognostic biomarker of PDAC. Therefore, cell function experiments were performed to verify its potential effect on pancreatic cancer. We firstly investigated the expression of HELLS in pancreatic cancer cell lines and normal human pancreatic ductal epithelial cells (HPDE). The results showed that the protein levels of HELLS were highly upregulated in PANC-1 cells than in HPDE cells (Fig. 7A). To determine the oncogenic roles of HELLS in pancreatic cancer, we stably knocked down HELLS in PANC-1 cells, and verified at mRNA and protein levels (Fig. 7B). The results showed that the downregulation of HELLS significantly inhibited cell proliferation (*P* < 0.05, Fig. 7C), and dramatically impaired the ability of the colony formation (Fig. 7D). Thus, we further investigated cell cycle by flow cytometry to examine whether HELLS expression affected cycle distribution in pancreatic cancer (Fig. 7E). As shown in Fig. 7F, 54.16 ± 1.568% of shHELLS-1, 47.73 ± 1.898% of shHELLS-2, 55.81 ± 2.127% of shHELLS-3, and 34.52 ± 1.817% of shHELLS-con were in S phase. The statistical data showed a significant increase in S fraction in shHELLS group compared with the shHELLS-con group (****P = 0.0001), indicating that knockdown of HELLS inhibited cell proliferation by arresting cells at S phase in pancreatic cancer.

## Discussion

SMARCA1 is a tumor-suppressor gene located on chromosome X [17]. Until now, the function of SMARCA1 in the type of cancers has not been a focus of research. Little was known about the physiological functions of SMARCA1 in pancreatic cancer. In our study, conflicting findings of the roles of SMARCA1 in pancreatic cancer were observed. On the one hand, high SMARCA1 mRNA expression was observed in pancreatic cancer, and SMARCA1 mRNA expression was remarkably correlated with tumor stages. On the other hand, high mRNA SMARCA1 expression was significantly correlated with favorable OS in patients with pancreatic cancer. Therefore, further studies consist of larger sample sizes worth to validate SMARCA1 expression patterns in patients with pancreatic cancer and to explore the roles of SMARCA1 in tumorigenesis.

SMARCA2 (also known as BRM) is one of two evolutionarily conserved catalytic ATPase subunits of SWI/SNF complexes sharing a high degree of amino acid sequence identity with SMARCA4 and interacting with transcription factors and other cellular proteins to modulate transcription activity of multiple genes [18]. Wilson BG et al. has demonstrated a compensatory role between SMARCA2 and SMARCA4, that a reciprocal assembly of SMARCA2 into SWI/SNF complexes when SMARCA4 is genetic inactive, providing insight into the mechanisms driving tumorigenesis [19]. SMARCA2 plays important roles in cell proliferation, linage specification and development, cell adhesion, cytokine responses, and DNA repair, which was previously implicated in risk and prognosis in lung, esophageal, colon cancer, and pancreatic cancer [18, 20]. However, conflicting evidence about its roles in various type of cancers has remained. On one hand, frequent loss of SMARCA2 expression was observed in patients with lung cancer and gastric cancer. It correlates with cancer aggressiveness and poor prognosis, suggesting a suppressive role of SMARCA2 in these tumors [21, 22]. On the other hand, SMARCA2 overexpression was found in human epithelial ovarian cancer. Xu X et al. reported a relatively higher expression of SMARCA2 in cisplatin-resistant ovarian cancer cells, leading to a significant reduction in apoptosis indicative of resistance to cisplatin [23]. Similar to SMARCA1, conflicting observations about SMARCA2 were also found in our study. Therefore, further studies are worth to evaluate the precise roles of SMARCA2 in pancreatic cancer.

SMARCA3 (also known as Helicase‑like transcription factor, HLTF) is frequently observed in colorectal cancer (CRC) and is negatively associated with the progression of CRC [24]. Consistent with the conclusion, in our study, despite no statistically significant difference in mRNA between pancreatic cancer and normal tissues, mRNA expression of SMARCA3 was significantly correlated with tumor stages, and high SMARCA3 mRNA expression was positively related to longer OS in pancreatic cancer. Of note, protein expression of SMARCA3 were observed in pancreatic cancer tissues, but not in normal tissues. Given that, further verifications are required to determine the mRNA and protein expression of SMARCA3 between pancreatic cancer and normal tissues.

Studies have pointed to a role for human SMARCAD1 in genomic instability during the past few years, which can lead to cell death or cancer development in higher eukaryotes [25]. On the one hand, knockdown of SMARCAD1 resulted in a significant decrease in breast cancer cell proliferation and colony formation, mainly through a potent inhibition of STAT3 phosphorylation [26]. The conclusion was consistent with the study from Al Kubaisy et al. [27], which also suggested a possible tumor oncogenic role for SMARCAD1. However, the expression of SMARCAD1 in patients with bladder cancer was associated with an increased survival time [28]. Anti-oncogenic properties of SMARCAD1 was also shown in HCC [25]. These data suggested that SMARCAD1 as a tumor suppressor. Notably, the role of SMARCAD1 seems conflicting in pancreatic cancer. The previous study has indicated that SMARCAD1 is highly expressed in pancreatic cancer tissues and negatively correlated with survival time. Mechanistically, SMARCAD1 promotes pancreatic cancer cell growth and metastasis via activating Wnt/β-catenin-mediated EMT [29]. On the contrary, in our study, we found that SMARCAD1 expressed lower in pancreatic cancer tissues than that in normal tissues, and significantly associated with unfavorable OS.

In addition to SMARCA2, SMARCA4 is another exclusive catalytic subunit of SWI/SNF complexes containing a bromodomain and an ATPase domain essential for the modulation of SWI/SNF chromatin remodeling [3, 30]. A recent study indicated that SMARCA4 functioned as a bona fide tumor suppressor and cooperatd with p53 loss and Kras activation [31]. SMARCA4 is frequently mutated in multiple cancer types, including non-small cell lung carcinoma (NSCLC) (10–35%), Burkitt’s lymphoma (15%) and childhood medulloblastoma (5–10%), and occasionally mutated in pancreatic adenocarcinoma, ovarian clear cell carcinoma and melanoma [19, 32]. Jelinic P et al. revealed that inactivating mutations in SMARCA4 were associated with poor lung adenocarcinoma outcomes. Moreover, re-expression of SMARCA4 through electroporation in SMARCA4-null H1299 NSCLCs resulted in a dose-dependent suppression of cell growth [33]. As for SMARCA5, it was previously observed overexpressed in many malignant neoplasms. Jin et al. found overexpression of mRNA and protein in breast cancer, significantly associated with TNM stages, tumor size, high proliferation index, and poor OS [34]. Consistent with the above conclusions, Wang H et al. reported that survival rates among patients with gliomas exhibiting high SMARCA5 expression were much poor than that low levels [35]. These data suggested that SMARCA5 might be a novel prognostic biomarker in those cancers. SMARCAL1 is implicated in cellular DNA replication stress, such as stabilization of DNA replication forks and inhibition of genome instability and tumorigenesis induced by oncogenes [36]. In this report, Puccetti MV et al. biologically demonstrate that loss of SMARCAL1 profoundly suppressed Myc-driven B cell lymphomagenesis, suggesting that SMARCAL1 is could provide a therapeutic opportunity in Myc-driven malignancies. In our study, although high mRNA expressions of SMARCA4/5/SMARCAL1 were all observed in pancreatic cancer patients compared to normal tissues, no significant correlation was found between those three SMARCA members and tumor stages and OS.

Significant overexpression of HELLS has been found in various cancers, including medulloblastoma, hepatocellular carcinoma (HCC), and CRC. Literature from Law CT et al. reported that HELLS was remarkably overexpressed in HCC and positively correlated with aggressive clinicopathological features and poorer prognosis than patients with lower HELLS expression, which was further confirmed by reduced HCC growth and metastasis both in vitro and in vivo following the depletion of HELLS. Moreover, the inactivation of HELLS resulted in metabolic reprogramming and reversed the warburg effect in HCC cells [37]. Parallel to this finding, Zhang G et al. found that HELLS expression was highly expressed in glioblastoma and positively associated with glioma progression. The oncogenic roles of HELLS in glioblastoma are likely mediated through interactions with E2F3 and MYC [38]. The above observation revealed that HELLS is a key epigenetic regulator driving those tumors’ pathogenesis. In our study, bioinformatics methods were used to detect potential prognostic biomarkers for challenging pancreatic cancer. Consistently, significantly higher mRNA and protein expressions of HELLS were observed in pancreatic cancer tissues compared to normal tissues. Although HELLS showed no statistically significant association with patients’ tumor stages, HELLS overexpression is significantly correlated with the poor OS. In addition, downregulation of HELLS inhibits pancreatic cancer cells proliferation and colony formation, and induces cell cycle arrest in vitro.

In addition to SMARCA family members, a highly conserved core subunit (SNF5, also known as SMARCB1) is present in all known variants of the SWI/SNF complex [39]. It has been reported that its mutation can evoke powerful genome-wide downstream effects, which may be counteracted therapeutically [40]. INI1, encoded by SMARCB1, functions as a tumor suppressor. Loss of INI1 expression leads to oncogenic activation of EZH2 (an enzyme that catalyzes trimethylation of histone H3 lysine 27, H3K27me3), especially in epithelioid sarcoma [41]. Even though EZH2 has been pursued as a therapeutic target for several types of tumors, such as sarcoma, lymphoma, and malignant rhabdoid tumor (MRT) [42], there is still much unknown how SMARCB1/INI1 drives EZH2 or how SMARCB1 interacts with the potential targets in pancreatic cancer. It would be interesting to explore their underlying mechanism and potential roles further, which would undoubtedly provide insights into a valuable link between chromatin remodelelling and pancreatic cancer.

In summary, we systematically analyzed the expression and prognostic value of SMARCAs in pancreatic cancer. Functionally, the data demonstrated that HELLS plays vital roles in promoting pancreatic cancer progression, and serves as a poor prognostic biomarker for pancreatic cancer.

## Data Availability

The results were analyzed online and aggregated directly from multiple databases without relevant accession numbers. Direct web links of datasets: ONCOMINE, https://www.oncomine.org/resource/login.html (Registration is needed to login into the database); Gene Expression Profiling Interactive Analysis (GEPIA), http://gepia2.cancer-pku.cn/; Kaplan–Meier Plotter, http://kmplot.com/analysis/; The Human Protein Atlas (HPA), https://www.proteinatlas.org/; Metascape, http://metascape.org.

## Abbreviations

SWI/SNF: Switch/sucrose nonfermenting complex
GEPIA: Gene Expression Profiling Interactive Analysis
TCGA: The Cancer Genome Atlas
GTEx: Genotype-Tissue Expression
HR: Hazard ratio
CIs: Confidence intervals
HPA: Human Protein Atlas
GISTC: Genomic Identification of Significant Targets in Cancer
OS: Overall survival
DFS: Disease-free survival
GO: Gene ontology
BP: Biological processes
CC: Cellular components
MF: Molecular functions
KEGG: Kyoto Encyclopedia of Genes and Genomes
PMSF: Phenylmethanesulfonyl fluoride
TBST: Tris-buffered saline with 0.1% Tween-20
ATCC: American Type Culture Collection
DMEM: Dulbecco’s modified Eagle’s medium
FBS: Fetal bovine serum
qRT- PCR: Quantitative real-time PCR
CCK-8: Cell counting kit-8
PBS: Phosphate-buffered saline
PI: Propidium iodide
TPM: Transcripts per million
HPDE: Human pancreatic ductal epithelial cells
HLTF: Helicase‑like transcription factor
CRC: Colorectal cancer
NSCLC: Non-small cell lung carcinoma
HCC: Hepatocellular carcinoma
H3K27me3: Trimethylation of histone H3 lysine 27
MRT: Malignant rhabdoid tumor
